# Neutrophil count as the centerpiece in the joined association networks of inflammatory and cell damage markers, and neuroendocrine stress markers in patients with stable angina pectoris following stenting

**DOI:** 10.1371/journal.pone.0215209

**Published:** 2019-04-11

**Authors:** Tamás Horváth, Gyöngyi Serfőző, Ádám Györkei, Imre Földesi, Tamás Forster, Margit Keresztes

**Affiliations:** 1 Invasive Cardiology Unit, Centre of Cardiology, Medical Faculty, University of Szeged, Szeged, Hungary; 2 Department of Biochemistry, Medical Faculty, University of Szeged, Szeged, Hungary; 3 Synthetic and Systems Biology Unit, Biological Research Centre, Szeged, Hungary; 4 PhD School of Biology, Faculty of Sciences, University of Szeged, Szeged, Hungary; 5 Department of Laboratory Medicine, Medical Faculty, University of Szeged, Szeged, Hungary; 6 Centre of Cardiology, Medical Faculty, University of Szeged, Szeged, Hungary; Yonsei University Medical College, REPUBLIC OF KOREA

## Abstract

**Objective:**

The primary aim of this study was to examine whether markers of cell damage and of the psycho-neuroendocrino-inflammatory/immune (PNI) system could be associated in patients with stable coronary artery disease (CAD) on the next day following percutaneous coronary intervention (PCI).

**Materials and methods:**

Blood samples of 23 patients (18 men and five women, mean age 62.9 ± 10.6 years), were collected immediately before (pre-PCI), immediately after (post-PCI), and on the day following PCI (1d-PCI). Lactoferrin, LL-37 and interleukin-6 (IL-6) were assayed in plasma, in addition to cortisol and chromogranin A (CgA), as well as CK, ASAT and ALAT. Total and differential leukocyte counts were also analysed.

**Results:**

At all the three time points, the monocyte fractions, the monocyte-to-lymphocyte and the neutrophil-to-lymphocyte ratios and CgA levels were elevated. We detected significant peri-procedural changes in the plasma levels of our PNI markers: IL-6 (p<0.05), lactoferrin, LL-37 (both: p <0.0001), CgA, (p<0.05), and cortisol (p<0.01). On the first day after PCI, highly significant associations were found of ASAT with IL-6 and neutrophil count (both: r>0.75, p<0.0001), and of CgA with neutrophil count and monocyte count (both: r>0.79, p<0.0001); furthermore, cortisol was also associated with neutrophil count (r>0.7, p<0.0001).

**Conclusions:**

The findings suggest that myocardial damage could correlate not only with an inflammatory reaction but, via neutrophil count, also with increased level of stress in stable CAD after PCI. Furthermore, 1d-PCI neutrophil count may serve as an easy-to-obtain integrative PNI measure in stable CAD.

## Introduction

Mainly due to advanced interventional cardiology and improved prevention, the mortality rate of coronary artery disease (CAD) decreased considerably in the last decade. Still, myocardial infarction probably remains a major obstacle to human longevity also in the coming decade [1]. According to a recent model, development and progression of CAD are determined mostly by the atherosclerotic inflammatory events and by the subsequent arterial repair [2]. Following percutaneous coronary intervention (PCI) with stent implantation, a secondary inflammatory response is initiated, which is triggered by endothelial denudation and disruption, in addition to plaque rupture and myocardial ischemia-reperfusion injury [3, 4]. Peri-procedural myocardial injury still represents a frequent complication of PCI (even in uneventful cases) [5]. It is mostly related to myocardial ischemia, that is elicited primarily by plaque disruption (leading to side branch occlusion or distal embolization) or by coronary vasoconstriction; ischemia and reperfusion are associated with inflammatory reactions [5–6].

Neutrophils and monocytes/macrophages have fundamental roles not only in the process of coronary atherosclerosis but in the post-PCI inflammatory events and in reperfusion wound healing responses as well [3–4, 6–7]. Elevated counts of monocytes were observed after stenting [8]. In a biobank study, the monocyte-to-lymphocyte ratio (MLR) correlated best with cardiovascular mortality in coronary angiography patients [9]. Some results support the prognostic significance of the neutrophil-to-lymphocyte ratio (NLR); its pre-PCI ratio predicted restenosis and/or mortality in stable and in acute CAD and was independently associated with the extent and severity of CAD [10–12]. However, Bressi et al. found that it is not the pre-procedural, but the increased post-PCI NLR that could be associated with peri-procedural myocardial injury in stable CAD [13].

Stenting was reported to trigger rapid neutrophil activation with the release of oxygen radicals and granule contents (e.g. lactoferrin) in stable and unstable angina [14–16]. A special, less utilized inflammatory marker, LL-37 is secreted abundantly by activated monocytes and related macrophages in atherosclerotic lesions [17–18]. The inflammatory response of the post-PCI state is characterized also by the increased levels of the cytokine IL-6; surprisingly, even diagnostic coronary angiography led to elevated plasma IL-6 in stable angina [15, 19–20].

Creatine kinase level and the percentage of MB isoenzyme (CK-MB) are widely accepted markers for the diagnosis of peri-procedural necrosis [5]. Post-PCI CK-MB elevation was associated with an increased risk of death, furthermore, it could independently predict the risk of mortality [5, 21]. Recently, alanine aminotransferase/transaminase (ALAT) and, particularly, aspartate aminotransferase/transaminase (ASAT) have been reconsidered as valuable cell damage markers in acute CAD, since their elevated post-stenting values were associated with infarct size, as well as with microvascular obstruction a few days after ST-elevation myocardial infarction [22].

Increasing evidence has indicated that psychosocial stress and depressive symptoms are major risk factors for the development and prognosis of CAD [23–25]. In addition, angina pectoris and PCI could also lead to stress [26]. Psychological stress activates predominantly the sympathetic-adrenomedullary system and the hypothalamic-pituitary-adrenocortical (HPA) axis. Plasma chromogranin A (CgA) and plasma cortisol levels reflect the activity of the neuroendocrine-sympathoadrenal and the HPA system, respectively [27–28]. It has been shown that psychological stress could result in monocytosis, in activation of neutrophils and in enhanced IL-6 secretion in CAD, probably through beta-adrenergic receptor mechanism [23, 29–30].

Despite the evidence that psychological stress and inflammation could be linked to CAD and PCI, and that peri-procedural myocardial injury is partially due to secondary inflammation, the associations of inflammatory factors and cell damage markers with neuroendocrine stress markers following elective PCI remain largely unexplored. Therefore, our primary goal was to investigate possible correlations involving cell damage markers and some markers of the psycho-neuroendocrino-inflammatory (PNI) system on the next day after stenting in stable angina. Additionally, we followed the peri-procedural changes of the tested PNI markers.

## Methods

### Patients

18 men and five women (with mean age 62.9 ± 10.6 years, range 43–79) with stable coronary heart disease participated in our study. Patients were eligible if they had characteristic symptoms of stable angina with positive stress test; the main inclusion criterion was significant stenosis of at least two major coronary arteries shown by coronary angiography (significant diameter stenosis >50%) to avoid unifocal coronary atherosclerosis. Exclusion criteria were: bypass surgery, cardiogenic shock, acute inflammations or any immunological disorders, malignancy, immunosuppressive/anti-inflammatory medication (except low-dose aspirin), recent major trauma/surgery, impaired renal and liver function, and drug/alcohol abuse. Our patients did not have myopathies, muscle damage, neuromuscular diseases, neurological disorders, nor electrolyte (Na, K) problems.

We used the New York Heart Association functional classification to estimate the severity of heart failure (NYHA, grades I-IV). There were three (13%), four (17%), and five (22%) patients in NYHA groups I, II, and III, respectively, whereas the rest of the patients did not have heart failure. The patients were on average overweight (BMI: 28.8 ± 4.0 kg/m^2^), eight patients (35%) had diabetes mellitus (type 2), and two (9%) were current smokers.

All patients took aspirin, while antihypertensives and statins were given to 22 (96%) and 20 (87%) patients, respectively. In addition, 16 (70%) patients were administered proton pump inhibitors and nine (39%) had sedatives; none of them took antidepressants. Clopidogrel was given before and after PCI; all patients were administered heparin (70–100 IU/kg) during PCI.

Our patients granted informed written consent before their entry into the study. The study protocol was approved previously by the Medical Ethics Committee at the Medical Faculty of University of Szeged (Human Investigation Review Board, Ref. No: 49/B-125/2008, 2382). The investigation conformed to the principles of the Declaration of Helsinki.

### Blood sampling and laboratory assays

Blood samples were collected immediately before PCI (pre-PCI), immediately after PCI (post-PCI), and on the next day following PCI between 7 to 9 h a.m. (1d-PCI). (The mean duration of PCI procedures was about 45 min, with a range between 30 min and one hour.) Blood cell counts were quantified by standard protocols using an automatic cell counter. In order to assess peri-procedural myocardial cell damage, CK, ASAT and ALAT were assayed in 1d-PCI samples. ALAT and ASAT enzyme activities were measured using commercially available kits (Roche Hungary Ltd., Budapest, Hungary) on Roche Modular P800 analyzer. CK and CK-MB activities were determined by kits from Human GmbH (Wiesbaden, Germany) on Roche Modular P800 analyzer. The upper limit of reference values for the tested enzymes were 170 U/l (females) and 195 U/l (males) in case of CK, /CK-MB ratio: 6%/, whereas 31 U/l (both genders) for ASAT and ALAT. (CK-MB was determined only in case of elevated total CK. Due to technical difficulties, white blood cell differentials, CK, ASAT and ALAT values were not available for all patients.) Since no reference interval was available for the MLR, we used the ratio of the median values of the monocyte and lymphocyte reference ranges (counts) as reference: 0.19. Similarly, we defined the NLR as the median values of the neutrophil and lymphocyte reference ranges (counts) as reference: 1.86.

For the assessment of plasma levels of chromogranin A (CgA), cortisol, lactoferrin, LL-37 and IL-6, blood samples were collected into cooled (4°C) EDTA-Vacutainer tubes, and plasma aliquots were stored at -80°C until analyses. CgA concentrations were determined by a radioimmunoassay kit (CGA-RIA CT, CIS Bio International, Gif-sur-Yvette, France; intra- and inter-assay CVs: <7%), reference range: 23–153 ng/ml. Cortisol was assayed by radioimmunoassay (DSL-2100, Diagnostic Systems Laboratories, Webster, TX, USA; intra- and inter-assay CVs: <12%), reference range: 160–620 nmol/l.

Lactoferrin concentration was measured by our „in-house” ELISA kit, developed according to Antonsen [31]. In brief, we coated microplates with rabbit anti-human lactoferrin (3.7 mg/l; Dako, Denmark) overnight at 4° C. Following two washing steps, plasma samples were added in duplicates (in wells: ten-fold dilution), and incubated for 1 h at room temperature. Following five washing steps, incubation with anti-lactoferrin-peroxidase was performed similarly. We produced the conjugate from Dako antibody and horseradish peroxidase (Calbiochem, USA) according to Wilson and Nakane [32], using a 4000-fold diluted form. After several washes, the plates were developed with TMB (BD Biosciences, USA), and the reactions were stopped by 4 N sulfuric acid. We used human lactoferrin (Sigma, USA) as standard; reference range/median for plasma lactoferrin: 40-200/90 ng/ml [31]. LL-37 concentration was determined by an ELISA kit (HK321, Hycult Biotech Inc., Uden, the Netherlands; intra- and inter-assay CVs: <10%); reference range: 25–250 ng/ml. IL-6 was assayed using a high sensitivity ELISA kit (BMS213HS; Bender MedSystems GmbH, Vienna, Austria; intra- and inter-assay CVs: 4.95% and 6.0%, respectively);, reference maximum: 8.7 pg/ml.

### Statistical analysis

First, we calculated the necessary sample size to obtain clinically meaningful correlation coefficients of r ≥0.6 between variables of interest with a power of 80% and with 5% level of significance. Although the power analysis resulted in a sample size of n = 19, we enrolled 24 patients to compensate for potential dropouts. Since one patient was transferred to a county hospital before PCI, 23 participants were involved in our study. According to Cohen, the effect size of correlation coefficients (r) with value of 0.5 is large; thus, regardless of p-values, it is unlikely, that correlation coefficients ≥0.6 reflect false associations, even in case of multiple tests [33]. To examine the changes of stress markers, alterations of inflammatory and various white blood cell count parameters in response to PCI, we used repeated measurements of ANOVA. All tests were two-sided with a significance level of p<0.05. In case of significant differences between the tested time points, we carried out pairwise comparisons using Bonferroni adjustments. Variables that, according to skewness and kurtosis values, did not show normal distributions were log10-transformed before correlational analyses to achieve normality (1d-PCI values of NLR, MLR, ASAT, CK), and non-parametric analysis (Friedman’s two-way analysis by ranks and Wilcoxon signed ranks test) was employed for showing significant peri-procedural differences (total leukocyte count, NLR, MLR, IL-6). Variable values are presented as mean ± standard deviation (SD) in the text, tables, and in supplementary tables while as mean ± standard error (SE) for figures.

To investigate associations among stress-related, inflammatory—and cell damage parameters, we applied partial correlational analysis with power analysis using R package ppcor; adjustments for sex, age, BMI and diabetes were carried out. As CgA levels are affected by proton pump inhibitor intake and the NYHA stadium of the patient, these factors were also used as controlling parameters for the partial correlational analyses of this variable [34–35]. As by Bonferroni adjustments, truely important differences might be considered as non-significant, we did not adjust p-values for multiple comparisons when calculating our numerous partial correlational analyses [36–37]. Statistical analyses were conducted using R (version 2.13.2) and IBM-SPSS software (version 20.0, SPSS Inc., Chicago, IL, USA).

## Results

### Leukocyte counts, fractions, NLR and MLR

From the data of Table 1 it is apparent that the monocyte fraction considerably exceeded the maximal reference value at all time points, and particularly in the 1d-PCI sample (Table 1). In parallel, monocyte count approached the upper reference limit directly after stenting and superseded it on the day following PCI, while it increased significantly (0.59 ± 0.27 G/l vs 0.67 ± 0.28 G/l, p<0.05). The 1d-PCI MLR yielded a markedly elevated value of 0.42, approximately two-fold higher than its predefined reference value of 0.19. The 1d-PCI MLR was significantly higher than the pre-PCI value (0.33 ± 0.17, p<0.01); furthermore, the pre- and post-PCI MLR values were already considerably elevated ratios. In addition, we found enhanced NLR in all the three sample types, particularly on the next day following stenting (2.68 ± 1.56, predefined reference value: 1.86). Slight, but significant changes were detected in the total leukocyte count (pre-PCI/post-PCI: 6.02 ± 1.67 G/l vs 6.59 ± 2.28 G/l, pre-PCI/1d-PCI : 6.02 ± 1.67 G/l vs 6.77 ± 1.98 G/l; both: p<0.05). Normal values were observed for neutrophil counts and fractions, and for lymphocyte counts at all time points.

**Table 1:** Leukocyte counts, fractions, NLR and MLR of our stable CAD patients directly before (pre-PCI), directly after stenting (post-PCI) and on the next day (1d-PCI).

Leukocyte count (G/l)/fraction (%)	Pre-PCI	Post-PCI	1d-PCI	Ref. range
Total leukocyte count	6.02 ± 1.67	6.59 ± 2.28*	6.77 ± 1.98*	3.7–9.5
Neutrophil count	3.55 ± 1.43	3.80 ± 1.53	4.21 ± 1.68	1.7–6.1
Neutrophil fraction	57.69 ± 8.86	57.38 ± 9.54	60.94 ± 7.75	44–68
Monocyte count	0.53 ± 0.22	0.59 ± 0.27	0.67 ± 0.28*	0.2–0.6
Monocyte fraction	8.84 ± 2.74	8.92 ± 3.31	9.78 ± 2.12	5–7
Lymphocyte count	1.72 ± 0.49	1.85 ± 0.42	1.71 ± 0.47	1.0–3.2
Lymphocyte fraction	30.32 ± 8.95	30.46 ± 9.55	26.42 ±7.51	27–34
NLR	2.27 ± 1.42	2.15 ± 0.95	2.68 ± 1.56	1.86
MLR	0.33 ± 0.18	0.33 ± 0.17	0.42 ± 0.20**	0.19

NLR, neutrophil-to-lymphocyte ratio; MLR, monocyte-to-lymphocyte ratio.

Data are presented as mean ± SD; in case of pre-PCI values, n = 22, for post-PCI, n = 16, and for 1d-PCI, n = 21; (except for pre-PCI and post-PCI total leukocyte counts, n = 23).

p*<0.05 for total leukocyte count: pre-PCI/post-PCI and pre-PCI/1d-PCI and for monocyte count: post-PCI/1d-PCI and pre-PCI/1d-PCI; p**<0.01 for MLR: pre-PCI/1d-PCI.

### Inflammatory factors and stress markers

**Fig 1:**
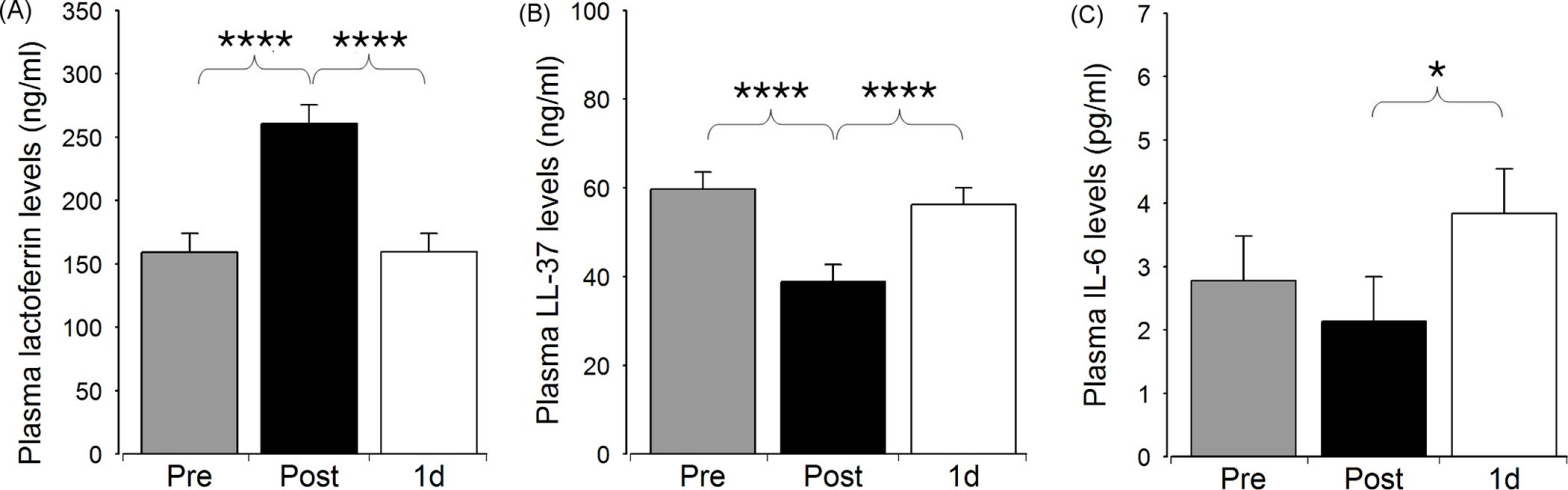
Plasma levels of the soluble inflammatory markers directly before (Pre), directly after stenting (Post), and on the next day following PCI (1d). Data are presented as mean ± SE, n = 23. Significant differences for lactoferrin and LL-37: Pre/Post, Post/1d - p****<0.0001; for IL-6: Post/1d - p*<0.05.

**Fig 2:**
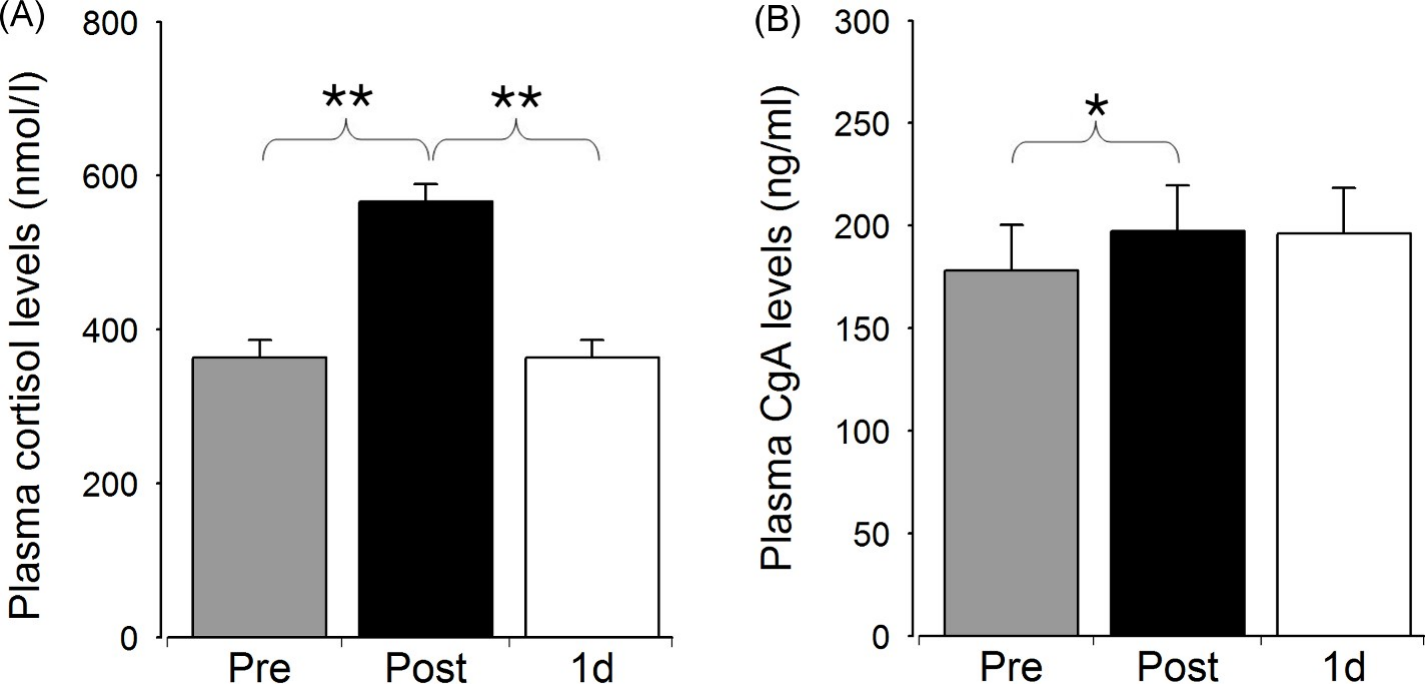
Plasma levels of the stress markers directly before (Pre), directly after stenting (Post), and on the next day following PCI (1d). Data are presented as mean ± SE, n = 23. Significant differences for cortisol: Pre/Post and Post/1d - p**<0.01; for chromogranin A (CgA): Pre/Post—p*<0.05.

We were interested in monitoring the changes in the plasma concentrations of the PNI markers following PCI. The concentrations of all the tested markers were altered directly after stenting with the exception of IL-6 (Figs 1 and 2, S1 Table). We observed a considerable (1.6-fold) elevation of plasma lactoferrin directly after PCI (159.31 ± 70.95 ng/ml vs 260.53 ± 80.76 ng/ml, p<0.0001), which was followed by a decrease to its initial level by next day (260.53 ± 80.76 ng/ml vs 159.35 ± 62.45 ng/ml, p<0.0001). Interestingly, the similarly highly significant changes of plasma LL-37 occurred in an opposite fashion: the post-PCI value was lower than the initial or the 1d-PCI value (38.90 ± 13.33 ng/ml vs 59.73 ± 18.26 ng/ml or vs 56.25 ± 14.58 ng/ml; for both: p<0.0001). By contrast, IL-6 concentration increased only on the next day following stenting (post-PCI/1d-PCI: 2.14 ± 2.06 pg/ml vs 3.84 ± 3.20 pg/ml, p<0.05). Concerning the stress markers, plasma cortisol elevated considerably directly after stenting (363.96 ± 108.86 nmol/l vs 566.65 ± 264.34 nmol/l, p<0.01), and returned to its initial level by the following day (566.65 ± 264.34 nmol/l vs 364.17 ± 100.93 nmol/l, p<0.01). Although only a slight increase of CgA was observed immediately after the intervention (178.13 ± 106.77 ng/ml vs 197.27 ± 131.34 ng/ml, p<0.05), it is to be noted, that all measured levels were above the reference range. Of all other values here, only the post-PCI lactoferrin exceeded the maximal reference concentration.

### Correlations among inflammatory factors, cell damage markers and stress markers on the next day following stenting

We were primarily interested in examining if, and to what extent the inflammatory process triggered by stenting could be associated with myocardial damage or with neuroendocrine stress response on the next day following intervention. The characteristic cell damage markers were assayed only on the next day after stenting; all values were in the normal ranges: ASAT: 29.80 ± 27.33 U/l (n = 20), ALAT: 27.38 ± 13.01 U/l (n = 21), and CK: 145.62 ± 172.64 U/l (n = 21). Only one patient was diagnosed with acute myocardial infarction (total CK over 800 U/l, and CK-MB ratio of 20.7%; ASAT: 137 U/l, ALAT: 33 U/l).

Concerning the correlations of the cell damage markers with the inflammatory markers, the associations of ASAT with neutrophil count (ng) and with IL-6 proved to be the most significant ones with high effect sizes (with r>0.760, p<0.0001, power>0.980). (The important multivariate-adjusted correlations are presented in Table 2, while all the examined ones are shown in S2 Table.) One of the stress markers, CgA showed also highly significant correlations with neutrophil count (ng) and, in addition, with monocyte count (mo) (both: r>0.790, p<0.0001, power>0.990). Similarly to CgA, also cortisol correlated strongly with neutrophil count (r = 0.730, p<0.0001, power>0.970). Not surprisingly, most inflammatory factors: ng, mo, NLR and MLR were highly associated (all: r≥0.780, p<0.0001, power>0.990), and the strongest correlations were observed between ng and IL-6 (r>0.840, p<0.0001, power>0.999). While ASAT was highly associated also with CK (r>0.740, p<0.0001, power>0.970) and slightly with ALAT (r>0.550, p<0.05, power>0.740), no significant associations could be found for ALAT (except with ASAT) (S2 Table). Two of the inflammatory markers, lactoferrin and LL-37, showed either no or only low-significance correlations (LL-37 with CK or mo: r>0.450, p<0.05, power>0.540) (S2 Table). The associations between ASAT and CgA or cortisol were relatively minor ones (both: r>0.500, p<0.05, power>0.620).

**Table 2:** Significant multivariate correlations between inflammatory markers, cell damage markers, and stress markers on the next day following stenting.

		ng	mo	(log) NLR	(log) MLR	IL-6	(log) CK	Cortisol	CgA
(log) ASAT									
	r	**0.762**^#^	0.471*	0.634**	0.541*	**0.779**^#^	**0.744**^**#**^	0.532*	0.501*
	p	**<0.0001**	0.046	0.002	0.016	**<0.0001**	**<0.0001**	0.019	0.031
(log) CK									
	r	0.636**	0.540*	0.533*	0.594*	0.471*	-	n.s.	0.548*
	p	0.001	0.013	0.015	0.004	0.039			0.034
Cortisol									
	r	**0.730**^**#**^	0.478*	0.628**	0.539*	0.630**	n.s.	**-**	0.500*
	p	**<0.0001**	0.035	0.002	0.013	0.001			0.017
CgA									
	r	**0.797**^**#**^	**0.793**^**#**^	0.565**	0.690***	0.441*	0.519*	0.500*	-
	p	**<0.0001**	**<0.0001**	0.008	0.0002	0.043	0.019	0.017	
ng									
	r	-	**0.798**^**#**^	**0.793**^**#**^	**0.780**^**#**^	**0.848**^**#**^	0.636**	**0.730**^**#**^	**0.797**^**#**^
	p		**<0.0001**	**<0.0001**	**<0.0001**	**<0.0001**	0.001	**<0.0001**	**<0.0001**
mo									
	r	**0.798**^**#**^	-	0.481*	**0.809**^**#**^	0.588**	0.540*	0.478*	**0.793**^**#**^
	p	**<0.0001**		0.034	**<0.0001**	0.005	0.013	0.035	**<0.0001**
(log) NLR									
	r	**0.793**^**#**^	0.481*	-	**0.832**^**#**^	0.636**	0.533*	0.628**	0.565**
	p	**<0.0001**	0.034		**<0.0001**	0.001	0.015	0.002	0.008
(log) MLR									
	r	**0.780**^**#**^	**0.809**^**#**^	**0.832**^**#**^	-	0.556**	0.594*	0.539*	0.690***
	p	**<0.0001**	**<0.0001**	**<0.0001**		0.01	0.004	0.013	0.0002

r, Pearson’s correlation coefficient; sp, statistical power; ng, neutrophil count; mo, monocyte count; NLR, neutrophil-to-lymphocyte ratio; MLR, monocyte-to-lymphocyte ratio. All parameters were adjusted for age, sex, BMI and diabetes; additional adjustments for chromogranin A: intake of proton pump inhibitors and grades of heart failure, n = 23 for correlations among cortisol, CgA and IL-6; n = 21 for correlations involving ng, mo, NLR, MLR, or CK; n = 20 for correlations of ASAT.

p*<0.05, p**≤0.01, p***≤0.001, p^#^≤0.0001 (bold fonts); (n.s.: not significant)

**Fig 3:**
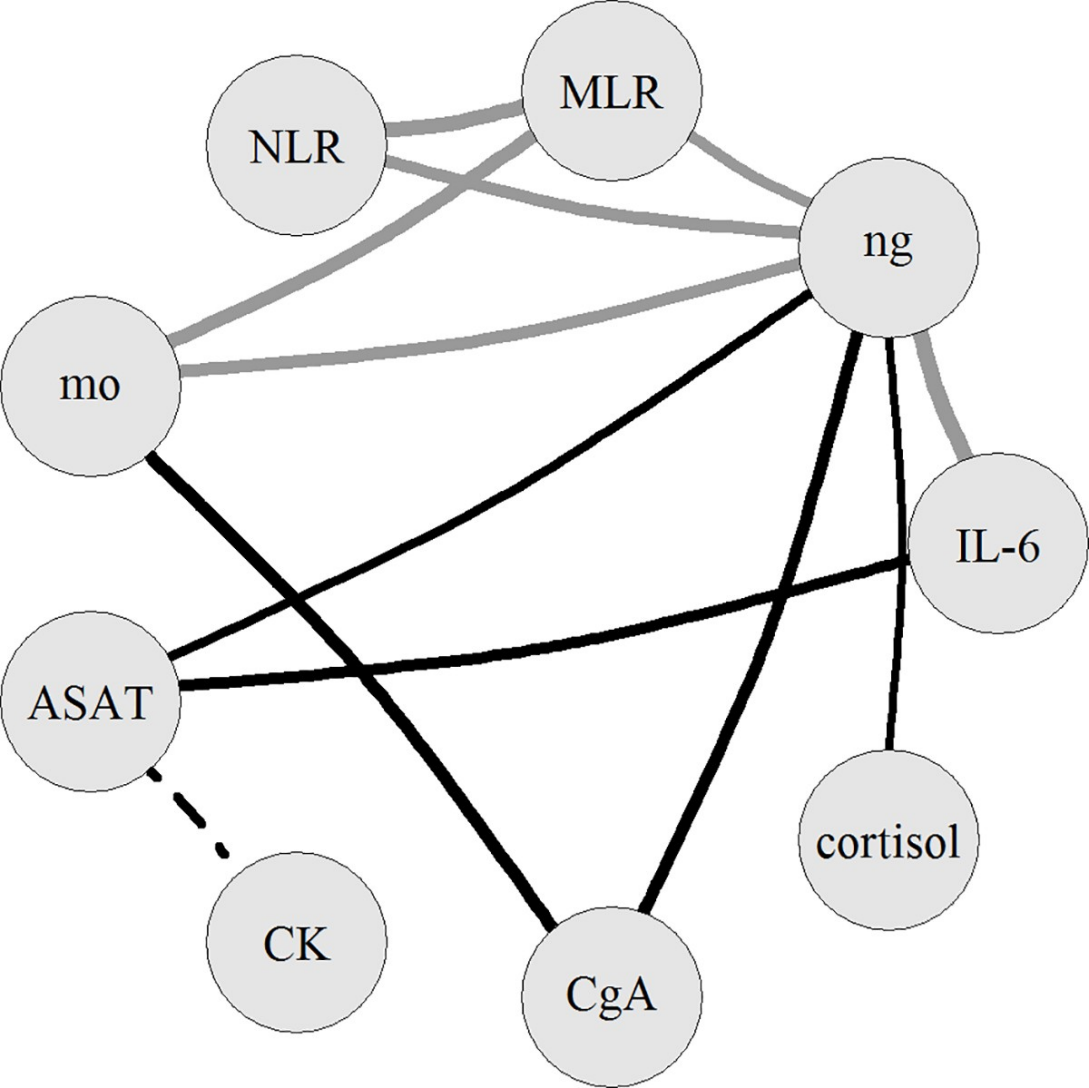
Major associations of inflammatory factors, cell damage markers and stress markers on the next day after stenting. ng: neutrophil count, mo: monocyte count, NLR: neutrophil-to-lymphocyte ratio, MLR: monocyte-to-lymphocyte ratio. Only correlations with r>0.7, p<0.0001 are shown, the thickness of a line is proportional to the value of Pearson’s correlation coefficient (r). A black line represents an association of an inflammatory factor with a cell damage factor or a stress marker; associations among inflammatory factors are shown in grey, while the correlation of cell damage factors with dashed line.

To visualize the predominant correlations found among the cell damage factors or stress markers and inflammatory factors, a graph was created (Fig 3). We found two major association networks (with r>0.750, p<0.0001), which include (1) ASAT, IL-6 and neutrophil count (ng) and (2) CgA, monocyte count (mo) and neutrophil count (ng); thus, these networks appear to be linked via ng. Looking for correlations r>0.7 (p<0.0001) of the components of these networks, we found two other associations involving (a) cortisol and ng, and (b) ASAT and CK. In addition, further, highly significant correlations of the inflammatory factors can be added to the picture: associations of ng with the related NLR and also with mo, MLR, and association of NLR with MLR (with r>0.750, p<0.0001).

## Discussion and conclusions

The main finding of our study is that myocardial cell damage is tightly associated with inflammation and, via neutrophils, also with enhanced level of stress in patients with stable CAD, on the day following stenting. During the correlational analysis of 12 parameters, two major association networks emerged: one that is composed of a cell damage marker (ASAT), a major inflammatory marker (IL-6) and neutrophil count, and an another one, which involves a marker of neuroendocrine-sympathetic activity (CgA), monocyte count and neutrophil count; these two networks are apparently linked by neutrophil count.

We observed elevated values of monocyte fractions, monocyte-to-lymphocyte (MLR) and neutrophil-to-lymphocyte ratios (NLR) both in the pre-PCI and post-PCI, and also in the 1d-PCI samples that probably indicates an inflammatory state. The increased level of initial MLR could reflect a severe atherosclerotic state, since the reciprocals of the pre- (and post-) PCI MLR values (1/0.33 = 3.03) are close to the critical lymphocyte-to-monocyte ratio of 3.1 that has been found in patients with high risk of severe atherosclerotic limb ischemia, which is frequently associated with CAD [38]. The slightly, but significantly elevated 1d-PCI monocyte count may indicate an inflammatory response to coronary wall injury caused by stent implantation [8]. Furthermore, the elevated MLR may also suggest a generally activated PNI system in our patients with stable CAD since, according to our prior and recent findings, high MLR could be associated not only with higher concentrations of inflammatory factors but with increased level of CgA and greater depressive symptom severity as well [39]. The augmented NLR may reflect the severity of CAD, and it could be associated with myocardial damage [10–11, 13].

All the tested inflammatory markers changed significantly during/after PCI, supporting the development of an inflammatory reaction initiated by the intervention. As lactoferrin is a specific and reliable activation marker of neutrophils, the significant increase of its post-PCI plasma level over the maximal reference value reflects substantial neutrophil activation triggered by the intervention [40]. Given the short lifetime of plasma lactoferrin (within 7 hours) [41]; the return of the increased plasma lactoferrin to its initial level by the next day following PCI indicates a rapid decrease in the activation state of neutrophils in the systemic circulation. Similarly, Gach et al. described a steep rise of plasma lactoferrin in coronary blood immediately after PCI in patients with unstable angina, which was reversed in 6 hours [15]; our results correspond also to our prior findings [42]. In contrast to these results, Aminian et al., could not detect a rise in plasma lactoferrin after stenting in stable and in acute CAD; given the high (over 250 ng/ml) initial values, this could have been due to overactivated and exhausted neutrophils [14].

LL-37, the only human cathelicidin, is regarded as a novel inflammatory marker [43]. It has a wide range of functions, including various antimicrobial and immunomodulatory effects, in addition to roles in apoptosis and wound healing [43]. It is produced primarily by different leukocytes; in atherosclerotic lesions, monocytes and related macrophages release LL-37 in an extensive manner [17–18]. Surprisingly, plasma LL-37 changed in an opposite fashion compared to lactoferrin: i.e. LL-37 level significantly decreased directly after PCI, but returned to the initial value by the following day. A similar rise of plasma LL-37 was observed one day after PCI in patients with acute myocardial infarction, who initially had lower plasma LL-37 than patients with stable CAD or control ones [44]. Since no differences were found between the local coronary values of the mentioned three groups, transient occlusion of the affected coronary arteries may hold a clue to variations in plasma LL-37 [44].

IL-6 is a multifunctional cytokine with a central role in inflammation, it is proposed as a major factor linking stress/depression and CAD [23, 30, 45]. Stenting induced a rise in plasma IL-6 on the next day of PCI, as has been described in unstable and in stable CAD patients and, in agreement with our previous findings [15, 19, 42]. It is to be noted, however, that even diagnostic coronary angiography could lead to enhanced IL-6 [19].

The increased plasma cortisol in the post-PCI samples presumably indicates a transient stress response initiated by the intervention that was downregulated by next day. (In a previous study, we could not show significant cortisol changes in stable CAD [42].) Enhanced plasma chromogranin A (CgA) levels may indicate a general neuroendocrine overactivity, including elevated sympathoadrenal activity [27–28]. However, since some medications (e.g. proton pump inhibitors) and cardiac failure could influence CgA concentration, the elevated pre- and 1d-PCI CgA concentrations cannot be interpreted clearly [34–35]. In contrast, the slight, but significant increase of CgA directly after stenting probably mirrored a PCI-triggered stress response.

On the first day after stenting, we found highly significant and strong correlations of ASAT with neutrophil count or IL-6, that could indicate an association of myocardial cell damage with inflammatory reaction. Lately, it became increasingly evident that stenting could frequently lead to ischemic myocardial cell damage, that is primarily caused by side branch occlusion or distal embolization by the disrupted plaques; this can bring about a secondary inflammatory response[5–6]. On the other hand, an inflammatory reaction is directly triggered by endothelial injuries and plaque ruptures, in addition to reperfusion [3–4]. Our results support the findings that neutrophils could have a key role in the ischemia-reperfusion injury [7, 16, 46]. Neutrophils are presumed to release stored IL-6 at local sites of inflammation, and they are known to have an important role in the orchestration and augmentation of the inflammatory process [47]. Recently, it has been shown that elevated neutrophil count (especially ≥ 8.355 G/l) could be an independent predictor of high thrombus burden and total coronary occlusion in patients with acute myocardial infarction [48].

The strong correlation of CK with ASAT supports the usefulness of the latter one as a cell damage marker after stenting in stable CAD. Although CK also showed correlations with neutrophil count and IL-6, surprisingly, these were less strong and significant associations than in the case of ASAT.

On the basis of the surprisingly strong correlation of CgA with neutrophil count or monocyte count, it is reasonable to suppose an association of the activity of the neuroendocrine-sympathoadrenal activity with the appearance of neutrophil granulocytes and monocytes in the circulation. According to investigations in Apoe^-^/^-^ mice, elevated sympathetic activity could directly lead to monocytosis via release of hematopoietic cells from the bone marrow (mediated by norepinephrine and beta3-adrenergic receptors) that is followed by increased monocytopoiesis in the spleen and by monocyte delivery to the blood stream [29, 49]. Therefore, the elevated values of monocyte count and MLR both before and after stenting may partly be due to sympathetic activation. Increased activity of the sympathetic system could also lead to increased neutrophil count (via both alpha- and beta- receptors), as shown in stressed rats [50]. We showed a remarkable correlation of cortisol with neutrophil count as well, although the related literature seems to be conflicting in this respect [50]. We found an additional correlation between of neutrophil count with monocyte count that could be reasoned by the parallel regulation of their release to the blood. Indeed, ischemia itself could lead to generation and recruitment of neutrophils and monocytes by granulocyte-macrophage colony-stimulating factor produced transiently by cardiac fibroblasts, as shown in human hearts after lethal infarct [51].

Since neutrophil count is shared between the two major association networks, hence, this raises the possibility that increased neuroendocrine-sympathetic activity (marked by CgA) could be involved in the peri-procedural myocardial damage by stimulating neutrophil release in patients with stable CAD. Our results indicate that increased cortisol might also be involved in this stress response. Leukocyte mobilization in stress is part of an ancient adaptation mechanism by which the body gets prepared to a possible attack or injury via releasing cells of the innate immune system to the blood, from where they can easily gain access to the site of acute challenge. Although neutrophils are indispensable in the elimination of foreign cells and cell debris, their uncontrolled, excessive activity could be noxious for the healthy cells nearby. A systematic review highlighted the importance of neutrophils in adverse clinical outcomes in acute coronary syndromes and/or after PCI or coronary artery bypass surgery [52].

While sympathetic activation may be a key factor in the release of neutrophils and in enhanced IL-6 secretion, (probably through beta-adrenergic receptor mechanism), increased vagal tone and heightened parasympathetic activity (e.g. by relaxation or vagus stimulation) could result in anti-inflammatory effects in CAD [53]. This is presumably due to a cholinergic pathway that suppresses pro-inflammatory cytokine release mediated by special nicotinic receptors [53–54]. Indeed, parasympathetic activity in the heart was found to correlate inversely with IL-6 in stable CAD (the Heart and Soul Study) [55]. Thus, it is plausible to suppose that improved parasympathetic-sympathetic balance could not only decrease neutrophil release to the systemic circulation but also reduce IL-6 production and acute phase reaction.

There are some limitations to be mentioned. First, the exact mechanisms underlying the correlations could not be clarified in this clinical study. Second, no further adjustments could be carried out in the multivariate analysis in addition to sex, age, BMI and diabetes (plus proton pump inhibitor intake and the NYHA stadium in case of CgA), in order to avoid model overfitting. Third, the sample size was small; however, the emerged, highly significant correlations had large effect sizes and thus support the validity of our results. Fourth, only one patient appeared to suffer peri-procedural myocardial infarction, thus it remains unclear, whether our findings could also be valid in a sample of patients with severe peri-procedural myocardial damage remains unclear. Fifth, we included patients with multivessel disease and we assumed that coronary artery stenosis of at least 50% of the vessel diameter in at least two coronary arteries reflects extensive atherosclerosis of the coronary system, although we did not use the sophisticated Syntax or Gensini scores.

In conclusion, our results suggest that activity of the stress-related sympathetic-neuroendocrine systems could correlate with neutrophil count that is associated with plasma levels of a major inflammatory cytokine (IL-6) and a cell damage marker (ASAT) in patients with stable CAD on the next day following PCI. Therefore, although not tested, our findings implicate the potential advantage of parasympathetic neuroimmunomodulation in the reduction of myocardial injury during/after stenting. Furthermore, neutrophil count may serve as an inexpensive integrative measure of the activated PNI network and the procedural cell damage after PCI in stable CAD. Further investigations are required to elucidate the details of PCI-related myocardial damage from PNI aspects and to elaborate PCI techniques with the least injuring consequences.

## Supporting information

S1 Table Plasma levels of inflammatory factors and stress markers directly before (pre-PCI), directly after PCI (post-PCI) and on the next day following stenting (1d-PCI). (DOCX)

S2 Table Multivariate correlations between inflammatory markers, cell damage and stress markers on the next day following stenting. (DOCX)
