# Supplementary material for: Neutrophil count as the centerpiece in the joined association networks of inflammatory and cell damage markers, and neuroendocrine stress markers in patients with stable angina pectoris following stenting
Source: PLoS One. 2019 Apr 11;14(4):e0215209. doi: 10.1371/journal.pone.0215209 (PMC6459524; doi:10.1371/journal.pone.0215209)
Supplement: S1 Table — (DOCX) [file pone.0215209.s001.docx]

**S1 Table. Plasma levels of inflammatory factors and stress markers directly before (pre-PCI), directly after (post-PCI) and on the following day (1d-PCI) of PCI.**

| **Blood plasma marker** | **Pre-PCI** | **Post-PCI** | **1-day-PCI** | **Ref. range** |
| --- | --- | --- | --- | --- |
| **Lactoferrin**  (ng/ml) | 159.31 ± 70.95 | 260.53 ± 80.76**** | 159.35 ± 62.45**** | 40-200 |
| **LL-37**  (ng/ml) | 59.73 ± 18.26 | 38.90 ± 13.33**** | 56.25 ± 14.58**** | 25-250 |
| **IL-6**  (pg/ml) | 2.78 ± 3.37 | 2.14 ± 2.06 | 3.84 ± 3.20* | <8.7 |
| **Cortisol**  (nmol/l) | 363.96 ± 108.86 | 566.65 ± 264.34** | 364.17 ± 100.93** | 160–620 |
| **CgA**  (ng/ml) | 178.13 ± 106.77 | 197.27 ± 131.34* | 196.13 ± 146.11 | 23-153 |

Data are presented as mean ± SD, n = 23. Significant differences are marked with asterisks:

p*<0.05 for IL-6: post-PCI/1d-PCI and for chromogranin A (CgA) pre-PCI/post-PCI;

p**<0.01 for cortisol: pre-PCI/post-PCI and post-PCI/1d-PCI;

p****<0.0001 for lactoferrin, pre-PCI/post-PCI and post-PCI/1d-PCI; and for LL-37, pre-PCI/post-PCI and post-PCI/1d-PCI.
