# Supplementary material for: Neutrophil count as the centerpiece in the joined association networks of inflammatory and cell damage markers, and neuroendocrine stress markers in patients with stable angina pectoris following stenting
Source: PLoS One. 2019 Apr 11;14(4):e0215209. doi: 10.1371/journal.pone.0215209 (PMC6459524; doi:10.1371/journal.pone.0215209)
Supplement: S2 Table — (DOCX) [file pone.0215209.s002.docx]

| **S2 Table. Multivariate correlations between inflammatory markers, cell damage and stress markers on the next day of stenting**. | | | | | | | | | | | | | |
| --- | --- | --- | --- | --- | --- | --- | --- | --- | --- | --- | --- | --- | --- |
|  |  | **log ASAT** | **ALAT** | **log**  **CK** | **Cortisol** | **CgA** | **ng** | **mo** | **log**  **NLR** | **log MLR** | **LF** | **LL-37** | **IL-6** |
| **log ASAT** | r | - | 0.560 | 0.744 | 0.532 | 0.501 | 0.762 | 0.471 | 0.634 | 0.541 | (0.078) | (0.107) | 0.779 |
|  | p | - | 0.011 | <0.0001 | 0.019 | 0.031 | 0.00001 | 0.046 | 0.002 | 0.016 | (0.769) | (0.686) | <0.0001 |
|  | pow | - | 0.743 | 0.979 | 0.687 | 0.621 | 0.986 | 0.557 | 0.873 | 0.705 | 0.056 | 0.066 | 0.991 |
| **ALAT** | r | 0.560 | - | (0.414) | (0.353) | (0.091) | (0.332) | (0.220) | (0.348) | (0.309) | (0.259) | (0.052) | (0.364) |
|  | p | 0.011 | - | (0.078) | (0.144) | (0.722) | (0.172) | (0.382) | (0.151) | (0.209) | (0.300) | (0.841) | (0.130) |
|  | pow | 0.743 | - | 0.460 | 0.341 | 0.061 | 0.305 | 0.151 | 0.332 | 0.266 | 0.195 | 0.050 | 0.361 |
| **log**  **CK** | r | 0.744 | (0.414) | - | (0.260) | 0.519 | 0.636 | 0.540 | 0.533 | 0.594 | (0.220) | 0.472 | 0.471 |
|  | p | <0.0001 | (0.078) | - | (0.298) | 0.019 | 0.001 | 0.013 | 0.015 | 0.004 | (0.383) | 0.038 | 0.039 |
|  | pow | 0.979 | 0.460 | - | 0.196 | 0.686 | 0.893 | 0.728 | 0.714 | 0.830 | 0.150 | 0.584 | 0.582 |
| **Cortisol** | r | 0.532 | (0.353) | (0.260) | - | 0.500 | 0.730 | 0.478 | 0.628 | 0.539 | (0.348) | (0.062) | 0.630 |
|  | p | 0.019 | (0.144) | (0.298) | - | 0.017 | <0.0001 | 0.035 | 0.002 | 0.013 | (0.126) | (0.799) | 0.001 |
|  | pow | 0.687 | 0.341 | 0.196 | - | 0.692 | 0.977 | 0.598 | 0.882 | 0.726 | 0.364 | 0.053 | 0.915 |
| **CgA** | r | 0.501 | (0.091) | 0.519 | 0.500 | - | 0.797 | 0.793 | 0.565 | 0.690 | (0.234) | (0.347) | 0.441 |
|  | p | 0.031 | (0.722) | 0.019 | 0.017 | - | <0.0001 | <0.0001 | 0.008 | 0.0002 | (0.320) | (0.127) | 0.043 |
|  | pow | 0.621 | 0.061 | 0.686 | 0.692 | - | 0.996 | 0.996 | 0.778 | 0.951 | 0.181 | 0.362 | 0.561 |
| **ng** | r | 0.762 | (0.332) | 0.636 | 0.730 | 0.797 | - | 0.798 | 0.793 | 0.780 | (0.305) | (0.397) | 0.848 |
|  | p | <0.0001 | (0.172) | 0.001 | <0.0001 | <0.0001 | - | <0.0001 | <0.0001 | <0.0001 | (0.216) | (0.094) | <0.0001 |
|  | pow | 0.986 | 0.305 | 0.893 | 0.977 | 0.996 | - | 0.997 | 0.996 | 0.994 | 0.260 | 0.425 | 0,9996 |
| **mo** | r | 0.471 | (0.220) | 0.540 | 0.478 | 0.793 | 0.798 | - | 0.481 | 0.809 | (0.384) | 0.456 | 0.588 |
|  | p | 0.046 | (0.382) | 0.013 | 0.035 | <0.0001 | <0.0001 | - | 0.034 | <0.0001 | (0.107) | 0.048 | 0.005 |
|  | pow | 0.557 | 0.151 | 0.728 | 0.598 | 0.996 | 0.997 | - | 0.604 | 0.998 | 0.400 | 0.549 | 0.819 |
| **log NLR** | r | 0.634 | (0.348) | 0.533 | 0.628 | 0.565 | 0.793 | 0.481 | - | 0.832 | (0.085) | (0.279) | 0.636 |
|  | p | 0.002 | (0.151) | 0.015 | 0.002 | 0.008 | <0.0001 | 0.034 | - | <0.0001 | (0.740) | (0.261) | 0.001 |
|  | pow | 0.873 | 0.332 | 0.714 | 0.882 | 0.778 | 0.996 | 0.604 | - | 0.999 | 0.059 | 0.222 | 0.893 |
| **log MLR** | r | 0.541 | (0.309) | 0.594 | 0.539 | 0.690 | 0.780 | 0.809 | 0.832 | - | (0.188) | (0.427) | 0.556 |
|  | p | 0.016 | (0.209) | 0.004 | 0.013 | 0.0002 | <0.0001 | <0.0001 | <0.0001 | - | (0.459) | (0.068) | 0.010 |
|  | pow | 0.705 | 0.266 | 0.830 | 0.726 | 0.951 | 0.994 | 0.998 | 0.999 | - | 0.12 | 0.487 | 0.761 |
| **LF** | r | (0.078) | (0.259) | (0.220) | (0.348) | (0.234) | (0.305) | (0.384) | (0.085) | (0.188) | - | (-0.156) | (0.277) |
|  | p | (0.769) | (0.300) | (0.383) | (0.126) | (0.320) | (0.216) | (0.107) | (0.740) | (0.459) | - | (0.515) | (0.235) |
|  | pow | 0.056 | 0.195 | 0.150 | 0.364 | 0.181 | 0.260 | 0.400 | 0.059 | 0.120 | - | 0.102 | 0.239 |
| **LL-37** | r | (0.107) | (0.052) | 0.472 | (0.062) | (0.347) | (0.397) | 0.456 | (0.279) | (0.427) | (-0.156) | - | (0.092) |
|  | p | (0.686) | (0.841) | 0.038 | (0.799) | (0.127) | (0.094) | 0.048 | (0.261) | (0.068) | (0.515) | - | (0.704) |
|  | pow | 0.066 | 0.050 | 0.584 | 0.053 | 0.362 | 0.425 | 0.549 | 0.222 | 0.487 | 0.102 | - | (0.064) |
| **IL-6** | r | 0.779 | (0.364) | 0.471 | 0.630 | 0.441 | 0.848 | 0.588 | 0.636 | 0.556 | (0.277) | (0.092) | - |
|  | p | <0.0001 | (0.130) | 0.039 | 0.001 | 0.043 | <0.0001 | 0.005 | 0.001 | 0.010 | (0.235) | (0.704) | - |
|  | pow | 0.991 | 0.361 | 0.582 | 0.915 | 0.561 | 0.9996 | 0.819 | 0.893 | 0.761 | 0.239 | 0.064 | - |

r, Pearson’s correlation coefficient; sp, statistical power; ng, neutrophil count; mo, monocyte count; NLR, neutrophil-to-lymphocyte ratio; MLR, monocyte-to-lymphocyte ratio. All parameters were adjusted for age, sex, BMI and diabetes; additional adjustments for chromogranin A (CgA): intake of proton pump inhibitors and grades of heart failure. n = 23 for correlations among cortisol, CgA, lactoferrin, LL-37 and IL-6; n = 21 for correlations involving ng, mo, NLR, MLR, CK or ALAT; n = 20 for correlations of ASAT.

Highly significant correlations with r>0.7, p<0.0001 are underlined, while the r and p values of the non-significant ones (at the level of α = 0.05) are shown in brackets.
